# Supplementary material for: A Likelihood Approach for Real-Time Calibration of Stochastic Compartmental Epidemic Models
Source: PLoS Comput Biol. 2017 Jan 17;13(1):e1005257. doi: 10.1371/journal.pcbi.1005257 (PMC5240920; doi:10.1371/journal.pcbi.1005257)
Supplement: S1 File — (TAR.GZ) [file pcbi.1005257.s014.tar.gz › HSPH_Online-SI-Revision/PF08/n1-mild/n1-mild_PF_table4.pdf]

1	R0	{1.27677, 7.89375}	{1.65862, 13.7658}	{{1.23033, 6.78948}, {2.23645, 19.9697}}	{31.7248, 82.5518}	inf	18.	46.9515	{2.62803, 109.744}	191.701	0.510456	newly	14.	19.26	{3., 38.}	79.1429	0.364289	Reff	0.891185	0.682886	{0.396942, 0.935554}	26.0376	ar	400.	755.69	{526., 979.}	88.9225
2	R0	{1.33231, 10.5259}	{1.75809, 14.6322}	{{1.26185, 8.26949}, {2.35155, 20.}}	{34.1934, 51.007}	inf	9.	28.6724	{5.64735, 63.1613}	230.695	0.424908	newly	6.	11.9	{4., 23.}	116.	0.301237	Reff	0.735435	0.818063	{0.526553, 1.14248}	24.9004	ar	467.	745.21	{474., 959.}	62.5075
3	R0	{1.2273, 6.76944}	{1.6527, 12.8216}	{{1.11228, 6.05643}, {2.29738, 19.0269}}	{38.9714, 97.4368}	inf	10.	53.5531	{3.04115, 116.125}	460.2	0.672732	newly	13.	24.03	{3., 51.}	120.538	0.418526	Reff	0.924157	0.833445	{0.454178, 1.08761}	27.356	ar	314.	587.94	{256., 863.}	98.2166
4	R0	{1.41215, 17.5742}	{1.58512, 13.9503}	{{1.20783, 7.0488}, {2.10096, 19.9315}}	{19.9863, 25.2403}	inf	22.	41.7159	{4.73903, 91.2966}	125.104	0.418442	newly	12.	18.05	{4., 34.}	84.25	0.309918	Reff	1.14808	0.708033	{0.412594, 0.956986}	38.3288	ar	465.	724.83	{508., 948.}	56.2344
5	R0	{1.42222, 6.15886}	{1.67996, 12.5797}	{{1.12977, 5.40945}, {2.22832, 18.9086}}	{28.5029, 111.365}	inf	19.	32.0593	{0.977135, 76.5433}	132.881	0.586359	newly	13.	13.74	{1., 33.}	81.5385	0.545579	Reff	0.797865	0.682248	{0.339082, 0.929439}	27.7965	ar	490.	606.39	{264., 857.}	45.0633
6	R0	{1.24363, 9.95596}	{1.68224, 14.7543}	{{1.25608, 8.70357}, {2.34498, 20.}}	{35.9278, 55.2102}	inf	38.	77.741	{24.6837, 146.954}	120.084	0.309031	newly	28.	34.98	{13., 61.}	55.8571	0.214028	Reff	0.93521	0.99889	{0.624508, 1.29507}	24.7938	ar	411.	769.38	{528., 991.}	87.4355
7	R0	{1.36478, 5.96056}	{1.6544, 12.7955}	{{1.13176, 5.72868}, {2.20345, 18.7697}}	{30.5996, 121.245}	inf	20.	51.9681	{3.63162, 125.877}	200.959	0.544933	newly	19.	22.87	{2., 49.}	76.6842	0.417628	Reff	0.848893	0.769566	{0.422794, 1.05101}	23.745	ar	484.	594.39	{294., 849.}	43.8905
8	R0	{1.36335, 17.6429}	{1.55691, 13.8097}	{{1.19672, 7.03382}, {2.03287, 20.}}	{21.1334, 26.4227}	inf	41.	29.8369	{1.35875, 63.5203}	58.488	0.53912	newly	19.	12.11	{1., 26.}	56.0526	0.541591	Reff	1.08932	0.679147	{0.414184, 0.884168}	37.6538	ar	484.	697.26	{495., 936.}	45.8884
9	R0	{1.26575, 5.28707}	{1.5794, 14.2296}	{{1.2158, 7.72003}, {2.18918, 19.9497}}	{26.7352, 169.615}	inf	24.	25.7317	{2.52418, 52.6365}	65.3034	0.393912	newly	28.	10.89	{1., 19.}	62.3929	0.614625	Reff	0.731603	0.665164	{0.387446, 0.867609}	19.9831	ar	472.	723.05	{521., 975.}	54.3326
10	R0	{1.42942, 9.49905}	{1.71267, 14.2923}	{{1.23792, 7.35283}, {2.40767, 20.}}	{26.6971, 61.0603}	inf	17.	30.9451	{1.61823, 61.3}	128.088	0.502519	newly	11.	12.43	{1., 24.}	71.	0.45054	Reff	0.773316	0.62371	{0.314789, 0.860103}	25.2441	ar	468.	769.24	{511., 1011.}	64.7564
11	R0	{1.46827, 15.0983}	{1.54712, 12.9024}	{{1.19407, 4.59684}, {1.9584, 20.}}	{17.7982, 30.2543}	inf	17.	23.2882	{0.162872, 57.1474}	116.299	0.705455	newly	6.	9.53	{0., 24.}	125.167	0.657076	Reff	1.17902	0.686139	{0.39117, 0.852673}	41.8044	ar	495.	682.43	{481., 901.}	40.3495
12	R0	{1.25751, 13.279}	{1.55252, 13.5598}	{{1.19988, 6.99025}, {1.95957, 19.9316}}	{25.5184, 30.6477}	inf	33.	29.899	{0.954477, 62.1713}	71.4922	0.586547	newly	20.	12.08	{0., 26.}	58.6	0.623922	Reff	1.01481	0.686652	{0.447238, 0.882975}	32.3369	ar	366.	695.6	{501., 918.}	90.0984
13	R0	{1.3425, 9.16361}	{1.66758, 12.7934}	{{1.10693, 6.0993}, {2.45236, 18.765}}	{33.3587, 55.4882}	inf	14.	16.5328	{0.217454, 39.612}	101.009	0.650064	newly	8.	7.07	{0., 15.}	82.875	0.703177	Reff	0.765225	0.611488	{0.295567, 0.817159}	27.9163	ar	442.	606.85	{316., 871.}	52.3281
14	R0	{1.18936, 6.20583}	{1.665, 12.7757}	{{1.11228, 5.72868}, {2.2983, 18.8228}}	{43.1827, 111.265}	inf	14.	32.7416	{0.977135, 76.5433}	185.744	0.575688	newly	10.	14.07	{1., 33.}	104.9	0.520922	Reff	0.752865	0.665054	{0.333577, 0.905931}	25.3011	ar	382.	599.41	{256., 863.}	71.0079
15	R0	{1.27137, 9.78868}	{1.70873, 14.2418}	{{1.20561, 8.3154}, {2.42513, 20.}}	{36.4207, 53.5077}	inf	28.	54.0373	{15.187, 111.017}	114.452	0.303472	newly	19.	24.58	{8., 45.}	62.7368	0.22679	Reff	0.827662	0.806343	{0.444235, 1.13593}	25.7155	ar	496.	760.79	{504., 1004.}	55.0423
16	R0	{1.23102, 13.6452}	{1.65384, 14.9746}	{{1.24055, 8.14745}, {2.18164, 20.}}	{35.0674, 28.4672}	inf	35.	43.1924	{5.5008, 85.7808}	68.696	0.343974	newly	16.	17.07	{3., 31.}	54.3125	0.283924	Reff	0.923265	0.675811	{0.391916, 0.921525}	28.6114	ar	445.	763.46	{563., 990.}	71.5775
17	R0	{1.27213, 13.7452}	{1.6291, 14.6786}	{{1.20744, 8.31818}, {2.15051, 19.9178}}	{30.5227, 26.6295}	inf	29.	63.4855	{15.0688, 140.199}	138.685	0.362148	newly	12.	27.28	{8., 51.}	141.333	0.351861	Reff	1.02661	0.784418	{0.450515, 1.04819}	25.1835	ar	367.	739.4	{479., 978.}	101.499
18	R0	{1.25318, 11.2151}	{1.56147, 14.0522}	{{1.1862, 6.99684}, {2.10133, 20.}}	{26.4409, 43.0099}	inf	28.	28.3114	{1.09677, 61.7627}	77.8023	0.551516	newly	18.	11.53	{1., 25.}	54.9444	0.557333	Reff	0.917328	0.669912	{0.417791, 0.897887}	27.2445	ar	416.	706.31	{503., 933.}	69.7861
19	R0	{1.46006, 8.11496}	{1.67848, 12.8575}	{{1.14096, 5.32461}, {2.24606, 19.0269}}	{26.9666, 71.9039}	inf	28.	39.5074	{1.37952, 79.2337}	104.545	0.536771	newly	24.	16.79	{2., 37.}	57.5417	0.489553	Reff	0.861435	0.666183	{0.382133, 0.878427}	27.0768	ar	496.	604.62	{256., 860.}	43.
20	R0	{1.14273, 6.71261}	{1.63168, 14.294}	{{1.19474, 7.40834}, {2.30431, 20.}}	{42.8884, 114.239}	inf	18.	42.8772	{8.65492, 98.4303}	159.733	0.395576	newly	18.	17.74	{5., 31.}	47.5556	0.257466	Reff	0.787341	0.687686	{0.360121, 0.921424}	23.1959	ar	362.	730.51	{495., 978.}	101.798
21	R0	{1.42432, 8.23905}	{1.72353, 13.4189}	{{1.28181, 5.39027}, {2.31117, 20.}}	{25.6504, 74.5163}	inf	21.	28.1179	{7.83131, 55.9545}	77.9769	0.262214	newly	16.	12.58	{5., 22.}	47.625	0.260945	Reff	0.914413	0.852529	{0.548389, 1.21845}	22.1638	ar	405.	739.51	{502., 941.}	82.7728
22	R0	{1.29386, 17.863}	{1.66188, 13.9933}	{{1.20336, 6.41748}, {2.38141, 20.}}	{31.4275, 27.3282}	inf	28.	48.8972	{5.64804, 102.315}	112.126	0.382908	newly	12.	20.26	{4., 35.}	98.6667	0.329721	Reff	1.06614	0.70686	{0.353244, 0.961694}	34.0381	ar	488.	734.35	{494., 995.}	51.8176
23	R0	{1.39274, 13.0167}	{1.62291, 14.4657}	{{1.20851, 7.36754}, {2.16634, 20.}}	{23.8799, 33.3005}	inf	35.	32.8729	{1.87692, 61.8449}	63.9086	0.464954	newly	27.	12.93	{1., 24.}	59.8889	0.567957	Reff	0.891354	0.677868	{0.401814, 0.943068}	26.49	ar	475.	743.12	{509., 966.}	57.3937
24	R0	{1.32178, 15.8991}	{1.57216, 13.9871}	{{1.19025, 7.3731}, {2.06589, 20.}}	{23.7528, 25.9258}	inf	48.	39.4691	{3.83097, 85.8391}	58.8375	0.419521	newly	20.	16.33	{3., 32.}	48.85	0.347663	Reff	0.896167	0.696515	{0.429969, 0.897794}	24.1317	ar	471.	715.04	{504., 948.}	52.6412
25	R0	{1.37568, 17.8427}	{1.66029, 13.7893}	{{1.1904, 6.98688}, {2.46068, 20.}}	{28.8224, 27.3315}	inf	27.	22.4044	{1.08438, 53.0341}	74.2423	0.578352	newly	11.	9.44	{0., 21.}	71.6364	0.58987	Reff	1.05377	0.629435	{0.295519, 0.85086}	40.2683	ar	398.	721.47	{491., 1001.}	81.3392
26	R0	{1.20993, 6.4656}	{1.64239, 14.7113}	{{1.20474, 8.2585}, {2.19451, 20.}}	{36.5482, 129.16}	inf	9.	37.62	{5.48718, 77.4396}	336.221	0.590107	newly	13.	15.43	{3., 30.}	67.1538	0.334164	Reff	0.834852	0.695618	{0.357346, 0.938388}	24.0786	ar	345.	731.32	{500., 993.}	111.977
27	R0	{1.20548, 13.8003}	{1.60521, 13.213}	{{1.2012, 6.40764}, {2.13013, 18.8606}}	{33.8277, 30.0157}	inf	30.	22.1648	{0.242939, 54.166}	70.0534	0.660261	newly	10.	9.21	{0., 21.}	70.5	0.593087	Reff	0.876384	0.650661	{0.376699, 0.839661}	26.6266	ar	350.	708.65	{481., 955.}	102.471
28	R0	{1.3176, 14.6594}	{1.52433, 13.6217}	{{1.18527, 6.41899}, {1.92076, 20.}}	{20.3157, 29.5844}	inf	22.	52.0505	{2.38401, 108.429}	170.726	0.506312	newly	16.	22.18	{3., 41.}	77.25	0.347576	Reff	1.11732	0.809366	{0.528182, 1.03316}	28.413	ar	362.	689.93	{473., 915.}	90.5884
29	R0	{1.28202, 6.23047}	{1.60187, 14.5939}	{{1.19607, 7.40799}, {2.27483, 20.}}	{27.7476, 135.974}	inf	11.	36.8831	{6.68584, 67.422}	252.425	0.502248	newly	14.	14.86	{3., 26.}	54.5714	0.309206	Reff	0.844851	0.687728	{0.359932, 0.932423}	22.8851	ar	391.	729.34	{487., 970.}	86.5934
30	R0	{1.28177, 8.83252}	{1.68033, 13.3145}	{{1.11461, 6.31117}, {2.47514, 19.1171}}	{37.172, 61.6188}	inf	22.	41.9536	{2.09512, 89.1508}	142.558	0.504778	newly	11.	16.9	{2., 34.}	101.455	0.405626	Reff	0.848532	0.707358	{0.344878, 1.02305}	28.5641	ar	427.	607.79	{272., 888.}	56.4754
31	R0	{1.35886, 5.97708}	{1.78722, 14.9864}	{{1.21548, 7.82661}, {2.42149, 19.9784}}	{35.6628, 152.53}	inf	14.	51.6405	{16.9377, 93.7797}	273.978	0.498964	newly	13.	22.27	{9., 38.}	85.7692	0.256352	Reff	0.712043	0.79391	{0.469527, 1.11576}	28.7952	ar	491.	784.6	{527., 1016.}	61.9348
32	R0	{1.27601, 17.9804}	{1.57952, 14.1171}	{{1.19261, 7.21488}, {2.10384, 20.}}	{26.3636, 25.7783}	inf	32.	39.7677	{0.487167, 88.4619}	79.6403	0.476119	newly	12.	16.15	{0., 32.}	82.9167	0.485297	Reff	1.03995	0.69779	{0.417133, 0.884544}	32.9014	ar	445.	709.56	{498., 936.}	60.0764
33	R0	{1.3368, 11.3235}	{1.68007, 14.4968}	{{1.22588, 7.72178}, {2.47834, 20.}}	{29.3643, 42.9012}	inf	41.	44.9592	{5.26191, 86.5494}	64.6684	0.364532	newly	18.	18.26	{4., 34.}	49.8889	0.296635	Reff	0.838174	0.681423	{0.316316, 0.93245}	24.7794	ar	417.	757.56	{535., 1017.}	81.6691
34	R0	{1.21021, 11.3635}	{1.62804, 13.5943}	{{1.19053, 7.98763}, {2.30177, 19.4331}}	{35.4813, 35.2289}	inf	19.	34.0849	{2.91362, 73.3453}	120.925	0.430718	newly	11.	14.54	{2., 32.}	81.2727	0.391677	Reff	0.958486	0.691396	{0.368639, 0.924557}	28.838	ar	327.	734.19	{518., 990.}	124.523
35	R0	{1.53382, 10.8866}	{1.61254, 14.6512}	{{1.19466, 7.68296}, {2.18031, 19.954}}	{20.6709, 46.8071}	inf	49.	46.651	{10.5801, 92.6629}	50.6965	0.317848	newly	34.	19.44	{6., 32.}	47.7059	0.369194	Reff	0.963239	0.713851	{0.406652, 0.96647}	27.3227	ar	476.	734.22	{515., 963.}	55.3487
36	R0	{1.20423, 6.83844}	{1.71483, 13.2969}	{{1.13239, 6.62607}, {2.42687, 19.0443}}	{45.0924, 99.8764}	inf	19.	41.3139	{2.09512, 112.742}	163.25	0.494046	newly	18.	17.68	{2., 41.}	69.7778	0.418945	Reff	0.714108	0.667949	{0.344878, 0.931291}	24.9731	ar	416.	614.97	{272., 873.}	63.3582
37	R0	{1.43629, 10.1548}	{1.61063, 14.2771}	{{1.19842, 7.82721}, {2.21123, 20.}}	{21.2559, 50.4341}	inf	18.	46.5347	{2.88379, 111.051}	184.713	0.462386	newly	19.	19.17	{3., 43.}	58.0526	0.335937	Reff	1.01977	0.716179	{0.363451, 0.943148}	29.9357	ar	367.	726.04	{489., 988.}	97.8311
38	R0	{1.37504, 8.93428}	{1.71098, 12.7196}	{{1.14292, 5.40945}, {2.42687, 19.0531}}	{31.9825, 56.7848}	inf	26.	39.3821	{1.37952, 101.319}	117.145	0.554091	newly	22.	16.97	{1., 41.}	65.3182	0.522754	Reff	0.764522	0.669825	{0.361212, 0.931781}	25.5058	ar	483.	618.11	{317., 867.}	46.0849
39	R0	{1.51637, 9.36742}	{1.66012, 13.0839}	{{1.11523, 6.0993}, {2.29887, 18.9086}}	{25.416, 54.6792}	inf	18.	27.9752	{1.00119, 65.5288}	115.584	0.526329	newly	14.	12.05	{1., 28.}	65.0714	0.501655	Reff	0.938633	0.6416	{0.334784, 0.861814}	34.3176	ar	441.	607.68	{311., 861.}	52.0907
40	R0	{1.23658, 9.82869}	{1.64605, 12.4059}	{{1.12977, 4.64686}, {2.20181, 18.4749}}	{37.3665, 48.6027}	inf	25.	42.2337	{1.34426, 101.22}	131.215	0.592706	newly	12.	17.81	{1., 40.}	102.083	0.478382	Reff	0.746894	0.710431	{0.414721, 0.958107}	22.5614	ar	414.	581.47	{256., 860.}	60.1184
41	R0	{1.24247, 5.4242}	{1.66501, 12.9565}	{{1.13239, 6.71734}, {2.22832, 18.8228}}	{37.7682, 140.565}	inf	10.	48.4479	{4.29022, 103.678}	411.57	0.635565	newly	10.	21.81	{3., 46.}	154.1	0.447444	Reff	0.861032	0.74544	{0.377161, 0.986173}	26.2863	ar	345.	616.68	{346., 854.}	85.513
42	R0	{1.28208, 4.40223}	{1.62941, 12.9119}	{{1.11523, 6.25107}, {2.20181, 18.834}}	{33.5783, 194.665}	inf	0.1	43.2234	{3.01878, 99.5981}	43124.8	2.34645	newly	0.1	18.52	{3., 37.}	18426.	2.073	Reff	0.847455	0.714087	{0.414721, 0.943795}	23.3658	ar	339.	581.33	{271., 858.}	82.9292
43	R0	{1.56773, 6.50474}	{1.83265, 13.2831}	{{1.13176, 8.14942}, {2.46976, 17.6755}}	{28.3696, 106.865}	inf	12.	57.3978	{6.77173, 109.743}	398.185	0.63672	newly	11.	25.22	{4., 51.}	153.636	0.41831	Reff	0.810516	0.675436	{0.33946, 1.03121}	30.1797	ar	498.	668.53	{356., 891.}	51.4357
44	R0	{1.10489, 6.01941}	{1.64128, 14.2741}	{{1.21938, 7.40869}, {2.27651, 20.}}	{48.5469, 139.456}	inf	6.	36.4193	{5.88305, 86.9532}	519.025	0.692982	newly	6.	14.97	{3., 30.}	172.167	0.445414	Reff	0.734752	0.656452	{0.357254, 0.856404}	20.9723	ar	346.	738.3	{540., 971.}	113.382
45	R0	{1.25888, 14.1926}	{1.60068, 15.4242}	{{1.21684, 8.16858}, {2.35147, 20.}}	{28.5894, 27.7785}	inf	40.	35.0197	{5.50949, 63.1648}	55.2732	0.362924	newly	16.	14.15	{3., 28.}	53.5625	0.349244	Reff	0.723856	0.677521	{0.330512, 0.924451}	22.5775	ar	471.	724.16	{504., 979.}	54.6794
46	R0	{1.45292, 16.616}	{1.62149, 14.5275}	{{1.21824, 7.10547}, {2.21328, 19.9598}}	{21.0833, 22.779}	inf	41.	57.6266	{13.4144, 104.412}	71.656	0.284492	newly	19.	23.53	{8., 42.}	56.0526	0.240226	Reff	1.11003	0.765483	{0.424139, 1.04433}	31.5959	ar	494.	748.39	{520., 990.}	52.747
47	R0	{1.22528, 16.6033}	{1.516, 13.9737}	{{1.1806, 6.98074}, {1.95978, 19.9436}}	{25.521, 24.7692}	inf	24.	46.2503	{6.76636, 98.5879}	123.841	0.399087	newly	5.	19.68	{6., 38.}	304.	0.568362	Reff	1.06599	0.775258	{0.484136, 0.985247}	27.886	ar	368.	679.81	{488., 911.}	84.731
48	R0	{1.1669, 6.92715}	{1.61648, 14.603}	{{1.22332, 8.08812}, {2.35201, 20.}}	{38.642, 112.212}	inf	8.	31.5382	{2.51585, 61.8745}	315.852	0.578276	newly	4.	13.26	{1., 28.}	255.5	0.535427	Reff	0.768987	0.669382	{0.31184, 0.877298}	21.2088	ar	416.	732.31	{519., 984.}	76.2476
49	R0	{1.32749, 16.0887}	{1.5448, 13.3023}	{{1.19932, 6.40105}, {1.96186, 19.742}}	{20.8884, 28.2477}	inf	24.	41.0943	{1.13813, 95.1397}	120.528	0.48412	newly	12.	17.52	{2., 34.}	90.3333	0.410371	Reff	1.11376	0.733454	{0.497768, 0.931766}	34.2424	ar	353.	700.4	{480., 896.}	98.4136
50	R0	{1.29636, 15.2609}	{1.61038, 14.0549}	{{1.18733, 7.34741}, {2.30094, 19.9456}}	{27.9097, 26.9183}	inf	49.	29.1958	{1.77628, 58.9032}	57.8691	0.56548	newly	23.	11.8	{1., 25.}	57.1304	0.586844	Reff	0.81541	0.653943	{0.310314, 0.882515}	23.4329	ar	470.	728.66	{511., 982.}	55.7277
